# Impacts of urban adaptation on reducing temperatures and heat-related deaths in Belgium

**DOI:** 10.1016/j.envint.2025.110039

**Published:** 2026-01

**Authors:** Fien Serras, Oscar Brousse, Charles H. Simpson, Inne Vanderkelen, Claire Demoury, Dirk Lauwaet, Nicole P.M. van Lipzig, Clare Heaviside

**Affiliations:** a Department of Earth and Environmental Sciences, KU Leuven, 3000 Leuven, Belgium; b Institute for Environmental Design and Engineering, University College London, WC1H 0NN London, United Kingdom; c Department of Meteorological and Climatological Research, Royal Meteorological Institute, 1180 Brussels, Belgium; d Risk and Health Impact Assessment, Sciensano, 1050 Brussels, Belgium; e Flemish Institute for Technological Research (VITO), 2400 Mol, Belgium

**Keywords:** Extreme heat, Urban heat adaptation, Population exposure

## Abstract

Extreme heat poses increasing risks to urban populations, yet the effectiveness of adaptation strategies at larger scales beyond neighbourhoods remains poorly quantified. The July 2019 heatwave in northwestern Europe, one of the hottest on record, offers a unique opportunity to evaluate city- and country-scale interventions in Belgium, in one of the world’s most urbanised mid-latitude regions. Using high-resolution urban climate modelling, we investigated the benefits of adaptation strategies on urban air temperatures. Our modelling of urban air temperatures suggests that the widespread deployment of cool roofs could reduce daily maxima by 2.1 °C, while combining them with a 20 % relative increase in vegetation could lower daily minima by 1.7 °C. Here, we found substantial temperature differences between compact and open urban areas and, subsequently, in population exposure to extreme heat. Furthermore, we estimate that the implementation of cool roofs could have avoided around a quarter of all heat-related deaths during this extreme heatwave event in Brussels. Our results show that adaptation strategies must be tailored to local urban characteristics to achieve substantial reductions in temperature and mortality, with more compact urban areas benefiting most from cool roofs during the day while the combination of cool roofs and increased vegetation has more impact on reducing nighttime temperatures in open urban areas.

## Highlights

• A quarter of heat-related deaths during July 2019's heatwave could have been prevented with adaptation strategies.
• A great fraction of the population is exposed to the highest temperatures, but these temperatures can be reduced the most.
• Urban morphology strongly affects cooling potential of heat adaptation strategies.

## Graphical abstract

**Figure:**
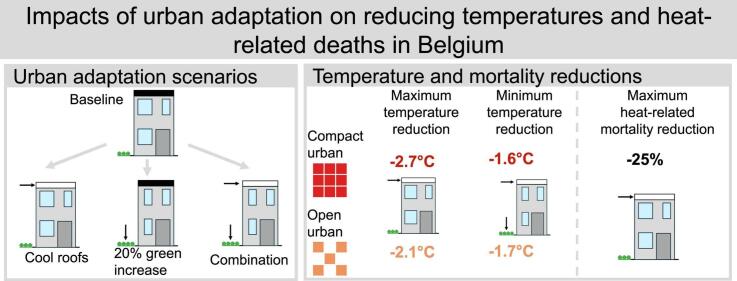

## 1 Introduction

Extreme heat is one of the key environmental threats to human health (Basu, 2002, Lüthi et al., 2023, Vicedo-Cabrera et al., 2021). Events with extreme heat are increasing in frequency, intensity and duration due to anthropogenic climate change (Perkins-Kirkpatrick and Lewis, 2020, Vautard et al., 2023). In recent years, Europe has experienced several continent-wide record-breaking heatwaves. For example, during the heatwave of July 2019, daily temperature records were broken in France, the Netherlands, Germany, the UK and Belgium (Sánchez-Benítez et al., 2022, Vautard et al., 2020). Such extreme temperatures have substantial impacts on mortality rates (Ebi et al., 2021, Kjellstrom, 2016). Estimates indicate that up to 70,000 people in Europe died during the summer of 2003 as a consequence of the higher summer temperatures (Robine et al., 2007). More recently, Ballester et al. (2023) estimated that more than 60,000 people died due to the extreme temperatures during the heatwave of 2022. While the July 2019 heatwave was the hottest heatwave in Belgium to date, with temperatures reaching 40 °C for the first time, no high-resolution, city-specific analysis on the increase in mortality has been carried out.

Under climate change, heatwaves are expected to become more frequent and intense (Fischer et al., 2021, Vautard et al., 2023). Additionally, overheating risks are exacerbated in cities due to the nature of the urban built environment (Wouters et al., 2017), a characteristic example being the urban heat island effect, whereby hotter temperatures are observed in cities compared with their rural counterparts (Oke, 1982). The urban morphology of cities, dependent on the land use and land cover, strongly affects local temperatures (Rahmani & Sharifi, 2025). Therefore, the Local Climate Zone (LCZ) classification system was proposed by Stewart & Oke (2012), whereby different urban environments are represented by one of ten different categories. The LCZs differ from each other depending on their density, height, building materials, and natural land covers. Additionally, the LCZ framework was found to be beneficial for understanding the link between urban climate and intra-urban health issues (Brousse et al., 2019).

To reduce risks induced by increased temperatures in cities (e.g. increased heat-related mortality (Ebi et al., 2021, Heaviside et al., 2016)), adaptation strategies have been devised to reduce air temperatures through alteration of the urban energy balance (Wang et al., 2022). Common heat interventions include the implementation of cool/reflective roofs, or increased greening. Urban climate models can simulate such adaptation strategies and provide insights into their expected cooling effect. For example, some studies modelled cool/reflective roofs with roof albedos ranging from 0.6 to 0.95 and obtained temperature decreases between 0.8 °C and 3 °C (Macintyre and Heaviside, 2019, Santamouris et al., 2011), with the differences mainly being explained by the implemented albedo, the considered period, the climate regime and the heat indicator. A review of modelling studies by Krayenhoff et al. (2021) found high albedo materials offered between 0.2 °C and 0.6 °C of cooling per 0.1 neighbourhood albedo increase in clear-sky conditions. Similarly, Azmeer et al. (2024) reviewed studies focusing on the influence of urban greenery and stated that the conclusions are strongly dependent on the type of greening, maintenance, the area of implementation, and the model resolution (Berardi et al., 2020). Several studies have estimated the impact of the temperature reductions on heat-related mortality (e.g. D. Chen et al., 2014, Heaviside et al., 2016, Macintyre and Heaviside, 2019, Simpson et al., 2024, Stone et al., 2014). They found that adaptation strategies such as cool roofs or increases in the urban vegetation fraction can reduce heat-related mortality by 5–32 %, though the magnitude strongly depends on the region, strategy, and modelling approach.

Here we extend existing studies by quantifying the population exposure to heat, as well as differences across urban morphologies, by applying 1 km resolution urban climate modelling using the Weather and Research Forecasting model (WRF, F. Chen et al. (2011)), with the Building Effect Parameterization coupled with the Building Energy Model (BEP-BEM, Martilli et al., 2002, Salamanca et al., 2010, Salamanca and Martilli, 2010) to simulate the effects of cool roofs and an increased vegetation fraction on near-surface air temperatures during Belgium’s hottest heatwave on record. We use the population density of Belgium to assess differences in heat exposure between the strategies across different urban environments characterised as Local Climate Zones (Stewart & Oke, 2012). Lastly, we estimate the number of avoidable heat-related deaths in Brussels based on the changes in maximum temperatures induced by the interventions.

## 2 Methods

### 2.1 Urban climate modelling with WRF

During the summer of 2019, Belgium was faced with three heatwaves, of which the heatwave between July 22nd and July 26th was the hottest. The official new Belgian temperature record of 39.6 °C was measured on the 25th of July and exceeds the previous record by 3 °C. The study period of this July 2019 heatwave was chosen as it is the most severe, though relatively short, heatwave of our measurements with an average daily maximum temperature of 33.8 °C (Koninklijk Meteorologisch Instituut van België, 2025).

We used the Weather Research and Forecast (WRF, F. Chen et al. (2011), version 4.5) regional climate model to simulate the hourly 2 m air temperature during the July 2019 heatwave in Belgium at a 1 km x 1 km horizontal resolution. WRF was coupled with the 3D Building Effect Parameterisation and its Building Energy Model (BEP-BEM, Martilli et al., 2002, Salamanca et al., 2010, Salamanca and Martilli, 2010) to have a detailed representation of the urban surface physics and to incorporate the impact of anthropogenic heat emissions. Through BEP-BEM, we account for the influence of the different urban areas on the momentum, heat and turbulent kinetic energy in cities (Martilli et al., 2002) as well as the exchanges of heat between the outdoor atmosphere and the indoor buildings (Salamanca et al., 2010, Salamanca and Martilli, 2010). We employed the Local Climate Zone (LCZ) characteristics to differentiate between different urban areas and the related urban morphology in the WRF model (Brousse et al., 2016, Demuzere et al., 2019, Stewart et al., 2014). The LCZs and urban morphological parameters were integrated in the WRF model following the Python version of the WUDAPT-TO-WRF strategy developed by Demuzere et al. (2022) based on work by Brousse et al. (2016). This strategy allows for the translation of the high-resolution (100 m) European LCZ map (Demuzere et al., 2021) to the model domain and simultaneously creates the non-urban (rural) scenario. Details on the urban morphologies can be found in Supplementary Table S1.

We used a two-way nesting strategy with 3 subdomains of respectively 12 km, 3 km and 1 km, the latter being used for our analyses with a gridsize of 252 by 324 and covering the whole of Belgium. This strategy allows for two-way feedback between domains and ensures a dynamically consistent multiscale dynamical downscaling. The outer domain was driven by ERA5 data at its boundaries (Hersbach et al., 2020). A map of the model domains can be found in Supplementary Fig. S1. All domains used the same physical and dynamical parameterisations we obtained from preliminary testing done over the two hottest days of the summer 2018: 26 and 27 July 2018. Our initial model setup was adopted from Brousse et al. (2023). The Kain-Fritsch convection scheme was activated for the first (outer) domain (Kain, 2004), and switched off for the second and third domains as they are at convection-permitting scales. We included the WRF single-moment 3-class microphysics scheme (Hong et al., 2004), the Dudhia shortwave and RRTM longwave schemes (Dudhia, 1989, Mlawer et al., 1997). Furthermore, we used Noah-MP over four soil layers in its default parameterisations as the land surface scheme (Niu et al., 2011, Yang et al., 2011). The model top was set at 50 hPa with 5000 m additional damping, the atmosphere was subdivided into 56 vertical layers. For the Planetary Boundary Layer parameterisation and the related Surface Layer scheme we used the Bougeault-Lacarrére (Bougeault & Lacarrere, 1989) with revised MM5 surface layer scheme (Jiménez et al., 2012). Following a sensitivity analysis, the Planetary Boundary Layer parameterisation scheme and the Surface Layer scheme were changed from the Bougeault-Lacarrére with revised MM5 to the Mellor-Yamada-Janjic scheme (Janjić, 1994) with the Eta Similarity Scheme (Janjić, 1994, Janjić, 1996, Janjić, 2002, Monin and Obukhov, 1954). Additionally, the use of adiabatic q was switched on.

### 2.2 Model evaluation

We ran our final model setup over the period from the 20th of July until the 26th, with the 20th and 21st being spin-up days not included in the final analysis. We evaluated our model against three sets of independent observations: 13 Automatic Weather Stations (AWS), 104 stations from the crowdsourced Weather Observation Website (WOW) and data from 971 personal Netatmo weather stations, after the latter two were validated using Crowd QC+ (Fenner et al., 2021). WOW and Netatmo stations were only considered when present in urban areas. No such selection was made for the AWS stations as only two were located in urban areas. We found RMSE values of 3.14 °C, 2.99 °C and 2.81 °C for AWS, WOW and Netatmo, respectively. For MAE, we found respective values of 2.48 °C, 2.43 °C and 2.34 °C; for Pearson’s R2, 0.87, 0.89 and 0.87; and for mean bias, 1.81 °C, 1.94 °C and 1.35 °C. This indicates that the baseline scenario generally overestimated the air temperatures. Therefore, we decided on bias correcting the maximum temperature before applying the temperature-mortality relationship (see Section 2.4. Heat-mortality relationship for Brussels).

Following recommendations from Brousse et al. (2023), a spatially explicit bias correction method was used for the daily maximum of the inner domain. We used three observational sets: WOW, Netatmo, and WOW combined with Netatmo. AWS observations were not included since these stations are not located in urban areas. We trained four different regressors (linear regression, ridge, Lasso and Random Forest Regressor) on the different observation sets where 6 urban morphological parameters were used as predictors: the urban fraction, the building surface to plan area fraction, the plan area fraction, the frontal area fraction, the surface height and the average building height. Following hypertuning of the model parameters using Grid Search CV library in Python and after testing the performance of the different regressors through a bootstrapping procedure where 80 % of the PWS were used for training the model and 20 % were kept for evaluation, we chose to employ the Random Forest Regressor combined with the Netatmo observational dataset for the daily maximum (Supplementary Table S2 and Supplementary Fig. S2). The corrections of the daily maximum temperatures for the baseline scenario are given in Supplementary Fig. S3. We assumed that the modelled difference between the baseline scenario and the modelled adaptation scenarios remains the same and adjusted the other scenarios as such, following Gasparrini et al. (2017) and Vicedo-Cabrera et al. (2021).

### 2.3 Heat adaptation strategies

The Local Climate Zone (LCZ) classification system was proposed by Stewart & Oke (2012), wherein different urban environments are represented by one of ten different categories. The LCZs differ from each other depending on, amongst others, their paved fraction, surface albedo, built-density and building height. As summarised by Rahmani & Sharifi (2025), the different LCZs affect local temperatures, which has an impact on the effectiveness of different heat adaptation strategies. Altering these properties by changing the urban environment through climate-sensitive designs can help mitigate heat-related hazards and exposure to higher temperatures in cities (Brousse et al., 2024, Krayenhoff et al., 2021). Additionally, the LCZ framework was found to be beneficial for understanding the link between urban climate and intra-urban health issues (Brousse et al., 2019).

To study the impact on heat and subsequently mortality, different heat adaptation scenarios were incorporated by modifying the parameters within each LCZ category. The effect of the presence of the city on local meteorology was quantified by including a theoretical rural scenario. In this scenario, the land use at every pixel was changed towards the dominant land use of the surrounding rural pixels using the Python version of WUDAPT-TO-WRF (Demuzere et al., 2022). We included a baseline scenario, to represent the cities as it was at the time of the heatwave. Details on the albedo and paved fraction of each LCZ can be found in Supplementary Table S1, and spatial details on the albedo and paved fraction of the baseline scenario can be found in Supplementary Fig. S4.

Subsequently, three different adaptation scenarios were implemented to study their effects on the air temperature in a city. In the first adaptation scenario, the albedo of all roofs of every LCZ was increased to 0.85 to represent cool roofs, following Azmeer et al., 2024, Sharma et al., 2016 and Simpson et al. (2024). In the second scenario, the fraction of urban vegetation (1 – Impermeable Surface Area (ISA)) in every urban pixel was increased by 20 %, following the 2050 goals of the Flemish National Adaptation plan (Departement Omgeving, 2022) which was applied to the whole of Belgium, since it was the most recent statement on adaptations at the time of the case study. This strategy was chosen since the general Belgian adaptation plan dates from 2010. Here we used equation (1) to calculate the ISA fraction (${\mathrm{ISA}}_{\mathrm{new}}$) that results from a 20 % relative increase in the unpaved urban fraction:

(1) $${\mathrm{ISA}}_{\mathrm{new}}=1-1.2*\left(1-ISA\right)$$

This equation was applied to every grid cell where the original ISA fraction exceeded 0.20, following the urban definition of Demuzere et al. (2022). A third scenario included the two abovementioned modifications.

### 2.4 Heat-mortality relationship for Brussels

To study the effects of heat on mortality and assess the benefits of adaptation strategies, we employed the temperature-mortality relationship developed by Crouzier et al. (2024) for Brussels over the period 1992 to 2019. This temperature-mortality relationship, estimated using quasi-Poisson regressions and distributed-lag nonlinear models (Gasparrini et al., 2010), gives the relative mortality risk associated with the daily maximum temperature. The risk of mortality reached its minimum for a daily maximum temperature of 24.0 °C and increased non-linearly above this. Similar to studies from Gasparrini et al. (2017) and Zhao et al. (2021), a long-term time series of temperature was used to determine the temperature-mortality relationship. From this relationship, the daily heat-attributable number of deaths can be derived (Gasparrini & Leone, 2014). We chose to use this relationship as we judged it was the most locally relevant for Brussels. Further details are provided in Crouzier et al. (2024). Using daily mortality data provided by the Belgian statistical office STATBEL over the heatwave period and the corresponding daily maximum temperature for Brussels, we calculated the potential heat-related mortality avoided by the different heat adaptation strategies as the difference between heat-related mortality in the baseline and in the adaptation scenarios. Because the temperature-mortality relationship is based on daily maximum temperature, the avoided mortality was calculated for the maximum temperature only.

## 3 Results

### 3.1 Modelled air temperature during the July 2019 heatwave

To gain insight into the effectiveness of urban adaptation strategies during heatwaves, we compared the near-surface (2 m) air temperatures of five different model simulations of the July 2019 heatwave in Belgium, which occurred from the 22nd to the 26th with WRF BEP-BEM (see Methods). The ‘baseline’ scenario represents the conditions in Belgium during the July 2019 heatwave. Subsequently, we incorporated three adaptation scenarios: (1) cool roofs, where the roof albedo was increased to 0.85 across all urban areas; (2) ‘green20′, with a 20 % relative increase in the urban green vegetation; and (3) a combined ‘green20 + cool roofs’ scenario. Lastly, to estimate the impact of cities on the local climate, we use a ‘rural’ scenario, in which the heatwave is simulated without the presence of cities (replacing urban categories with rural land use categories). We focus on the maximum and minimum air temperatures in urban areas, because of their well-studied relationship with human comfort and mortality (e.g. Ballester et al., 2023, Basu, 2002, Gosling et al., 2009 and Petkova et al. (2014)). Urban areas are defined as areas where the paved fraction is higher than 0.2, following Demuzere et al. (2022). Additionally, we use the urban classification of the baseline scenario across the analyses of the other scenarios.

**Fig. 1:**
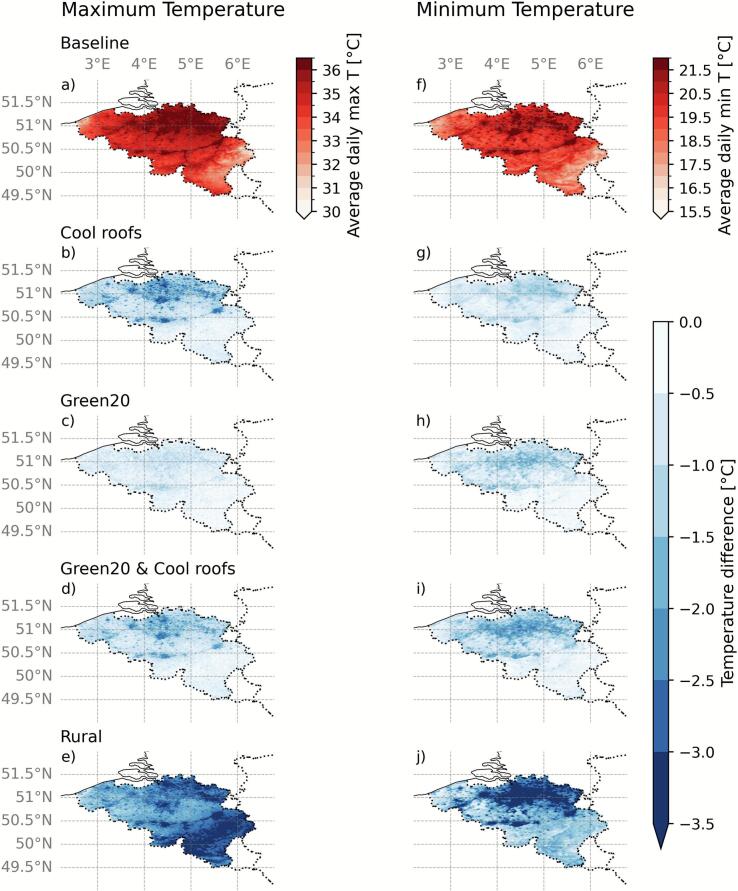
**Spatial distribution of maximum and minimum air temperatures in the scenarios**. Daily average maximum and minimum air temperatures for three adaptation scenarios during the July 2019 heatwave: the baseline, cool roofs, a 20% relative increase in urban vegetation, a scenario that combines the cool roofs with the 20% relative increase and a non-urban ‘rural’ scenario. Panels a until e relate to the daily maximum temperature. Panels f until j represent the daily minimum temperatures, a and f show the absolute temperature in the baseline scenario. b and g, c and h, d and i and e and j represent the temperature difference of the cool roofs, increased green fraction, the combined scenario and the rural scenario, respectively. (For interpretation of the references to colour in this figure legend, the reader is referred to the web version of this article.)

The implemented adaptation scenarios lead to different albedos, paved fractions and resulting air temperatures (Table 1) and affect the spatial temperature patterns (Fig. 1), with differences up to 3.6 °C. Large temperature differences were observed in the more urbanised northern part, which we attributed to the increased surface roughness, which importantly decreased the wind speed compared to the rural scenario (Supplementary Fig. S5). Similarly, the rural scenario had more clouds over the southern part of Belgium compared to the baseline scenario, partially causing the large differences in maximum temperatures as observed in Fig. 1. However, since few urban areas are located in these regions, we consider this to have a limited impact on our further discussions.

**Table 1:** **Summary of adaptation scenarios.** Overview of the average albedo, the average paved fraction, the daily average maximum and minimum air temperatures in urban areas (paved fraction > 0.2) for the baseline, three adaptation scenarios (cool roofs, a 20 % relative increase in urban vegetation, a scenario that combines the cool roofs with the 20 % relative increase) and the rural scenario, in compact urban areas (LCZ2 and LCZ3) and open urban areas (LCZ6 and LCZ9). The urban areas are based on the classification in the Baseline scenario. The temperature difference refers to the difference between the scenario and the baseline. LCZ8 is left out due to its low paved fraction combined with the low number of inhabitants, while other LCZs do not occur in Belgium.

Scenarios	Cities	Cool roofs	Relative 20 % increase in unpaved fraction	Average urban albedo [-]	Average urban paved fraction [-]	Average daily maximum temperatures [°C], (temperature difference [°C])			Average daily minimum temperatures [°C], (temperature difference [°C])		
						All urban areas	Compact urban areas	Open urban areas	All urban areas	Compact urban areas	Open urban areas
Baseline	X			0.24	0.40	36.15	36.48	36.18	21.72	22.94	21.74
Cool roofs	X	X		0.47	0.40	34.07 (−2.08)	33.75 (−2.73)	34.09 (−2.09)	20.77 (−0.95)	21.79 (−1.15)	20.79 (−0.95)
Green20	X		X	0.26	0.28	35.39 (−0.76)	36.12 (−0.36)	35.37 (−0.81)	20.57 (−1.15)	22.27 (−0.67)	20.52 (−1.22)
Green20 & Cool roofs	X	X	X	0.38	0.28	34.44 (−1.71)	33.92 (−2.56)	34.50 (−1.68)	20.05 (−1.67)	21.36 (−1.58)	20.02 (−1.72)
Rural				0.22	0	33.22 (−2.93)	33.34 (−3.14)	33.23 (−2.95)	18.14 (−3.57)	18.24 (−4.7)	18.16 (−3.58)

Among the three adaptation scenarios, compared to the baseline scenario, the cool roofs scenario resulted in the largest reductions in average daily maximum air temperatures, while the combined ‘green20 + cool roofs’ scenario led to the largest reductions in the average daily minimum air temperatures, regardless of the type of urban area. The average reductions by cool roofs in urban areas during the heatwave were 2.1 °C for daily maximum temperatures and 0.9 °C for daily minimum temperatures. In contrast, the green20 scenario produced less cooling for the daily maxima (0.8 °C), but larger reductions in minimum temperatures (1.1 °C) compared to the cool roofs. The combined scenario achieved intermediate results for maximum temperatures (1.7 °C), but outperformed both individual scenarios for minimum temperature reduction (1.7 °C). Besides, the presence of cities and urban areas in Belgium contributed 2.9 °C to the maximum temperatures and 3.6 °C to the minimum temperatures. Overall, cool roofs mostly impacted daytime temperatures, while combining cool roofs with an increased vegetation fraction led to the largest reductions in nighttime temperatures.

### 3.2 Population exposure to heat

**Fig. 2:**
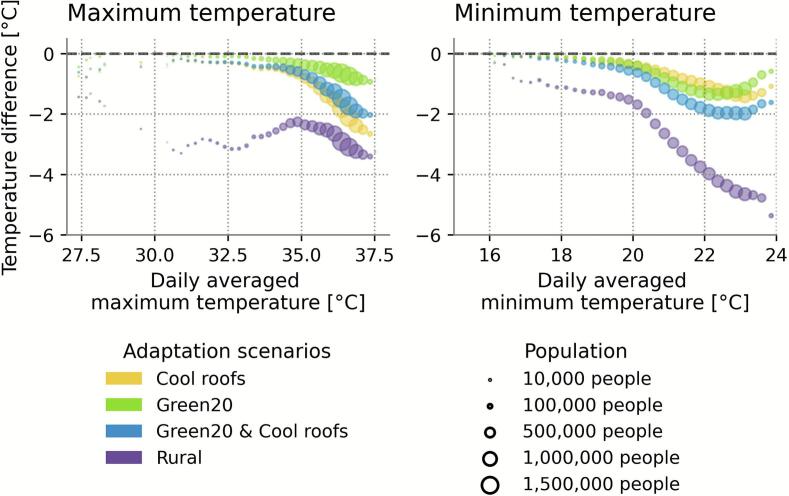
**Reductions in population exposure to heat.** Reduction of average daily maximum (left) and minimum (right) temperatures induced by different scenarios, compared to the baseline scenario. The size of the dots represents the fraction of the Belgian population experiencing baseline temperatures (x-axis) during the July 2019 heatwave, grouped per 0.25 °C. The average daily maximum (left) and average daily minimum are shown.

We assessed the distribution of simulated daily maximum and minimum air temperatures experienced by the Belgian population during the heatwave (Fig. 2). The majority of the urban population was exposed to relatively high maximum and minimum temperatures (50 % or 3,654,997.5 people to 36.1 °C or 21.8 °C respectively, or higher, Supplementary Table S3). Compared to the baseline scenario, heat interventions reduced heat exposure.

The magnitude of the reductions increases with higher maximum temperatures. This pattern reflects the spatial targeting of interventions and related mechanisms. Cool roofs were the most effective in compact city centres, where the maximum temperatures were the highest, as observed in Fig. 1. The high building density in city centres coincides with a large roof area available for cool roofs. By reflecting more incoming solar radiation, less energy remains to warm the surroundings. Due to the low green fraction in the city centres, a relative increase in greenery does not lead to a large absolute increase. The rural case had more clouds over the southern part of Belgium, potentially affecting the pattern of the temperature difference (see Section 3.1 for more details). Both could explain the observed differences. By contrast, minimum temperatures exhibited a different pattern. The largest reductions did not coincide with the locations of the highest minimum temperatures but rather occurred on the outskirts of the city. Because these urban areas initially had a high green fraction, a 20 % increase resulted in a larger added green area than in the city centre, leading to the largest reductions in minimum temperature. Overall, these trends are consistent with the spatially-averaged results (Table 1), highlighting the different mechanisms influencing daytime and nighttime cooling.

Additionally, a small share of the urban population (1 % or 73,100.0 people) experienced lower average maximum and minimum air temperatures compared to the median population (33.1 °C, respectively 19.1 °C, see Supplementary Table S3). These individuals mainly live at the Belgian coast and the higher parts of southern Belgium, showing the cooling effect of the sea breeze and of higher orographic locations.

### 3.3 Differences across local climate Zones

Using Local Climate Zones (LCZs) to categorise the urban environment, we found that, in the baseline scenario, the compact urban areas (LCZ2, compact mid-rise and LCZ3, compact low-rise) were hotter compared to the open urban areas (LCZ6, open low-rise and LCZ9, sparsely built) (Table 1). The gap between compact and open morphologies was higher for minimum temperatures than for maximum temperatures in the baseline scenario (1.2 °C versus 0.3 °C, Table 1). To study the impact on the population, the remainder of this section focuses on the reductions in temperature for the median of the population.

**Fig. 3:**
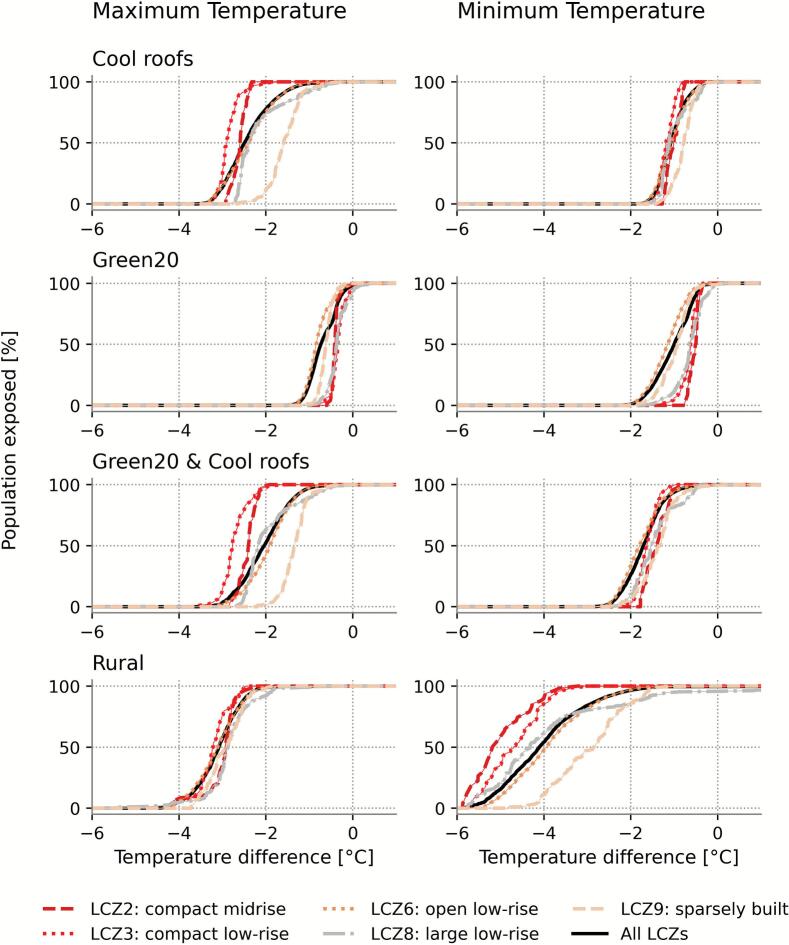
**LCZ-dependent distribution of population exposure to temperature differences under different scenarios, compared to the baseline scenario.** Cumulative distribution of the population within the different LCZs that experience the temperature difference (average daily maximum (left) and minimum (right)) for the rural and three adaptation scenarios compared to the baseline scenario. The black line represents the population over all LCZs.

Among all adaptation scenarios, cool roofs provided the strongest reduction in maximum temperatures. The effect was largest in compact LCZs, where the high proportion of roof coverage maximises their impact, and smaller in open LCZs, which have a smaller proportion of their area covered by roof. For at least 50 % of the population within each LCZ, cool roofs lowered the maximum temperatures by 2.6 °C for LCZ2 and 2.9 °C for LCZ3 compared to 2.4 °C in LCZ6 and 1.6 °C in LCZ9 (Fig. 3). By contrast, increasing urban greenery was most effective for reducing minimum temperatures. The temperature difference, also referred to as the cooling potential, scaled with the existing unpaved fraction due to the relative increase in greening: LCZ6, which already contains a substantial green cover, showed the greatest reduction in minimum temperature (1.2 °C), while the compact urban areas have less cooling (0.5 °C for LCZ2 and 0.6 °C for LCZ3).

Implementing the combined adaptation scenario further enhanced temperature reductions across all LCZs. Reductions in maximum temperatures ranged from 1.4 °C (LCZ9) to 2.8 °C (LCZ3), and minimum temperature reductions were between 1.3 °C (LCZ9) and 1.8 °C (LCZ6). The rural scenario, in which urban areas were replaced by non-urban land cover, revealed that the presence of the city has the highest impact on the temperatures: up to 3.2 °C (LCZ3) for maximum temperatures and 5.2 °C (LCZ2) for minimum temperatures. This underscores the strong role of urban morphology and heat storage in driving elevated minimum temperatures.

Across adaptation scenarios, the range in cooling potential is generally larger for maximum temperatures than for minimum temperatures (Fig. 3). Exceptions were found in compact LCZs and under the rural scenario, for which minimum temperatures show a stronger variation. The narrower ranges observed in LCZ2 and LCZ3 for all scenarios likely reflect their smaller spatial extent and more homogeneous morphology. For the minimum temperature, the largest variability occurred in the rural simulation, highlighting the impact of the urban morphology through the different LCZs. The majority of the Belgian population lives in LCZ6 (open low-rise) (5.5 M people, Supplementary Fig. S6). Meaning that, while the largest cooling occurred in the more compact LCZ2 and LCZ3, the majority of Belgian residents would benefit most from interventions in LCZ6, indicating that the importance of greenery should not be underestimated.

### 3.4 Reducing heat-related mortality in Brussels

**Fig. 4:**
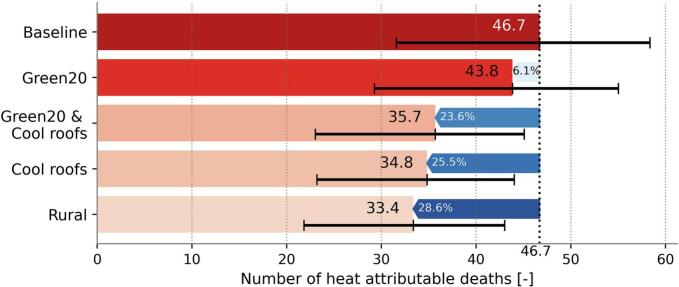
**Heat-related mortality under the different adaptation scenarios during the July 2019 heatwave in Brussels.** The number of heat-related deaths per scenario with the 95% Confidence Interval and the reduction relative to the baseline heat-related deaths are given.

During the 5-day July 2019 heatwave, a total of 112 all-cause deaths were observed in Brussels, Belgium’s capital, with 1.198.726 inhabitants (STATBEL, 2019). Of those 112 deaths, we estimate 46.7 (95 % confidence interval [31.6; 58.3]) to be attributable to high temperatures (Fig. 4). Of these, 33.4 [21.8; 43.0] deaths or 29.8 % of all deaths could be attributed to urban-induced heat, highlighting the additional burden imposed by the city’s built environment (Fig. 4). Our results indicate that a 20 % relative increase in green space could have reduced heat-related mortality in built-up areas by 6.1 %, preventing an estimated 2.9 [–23.5; 29.1] deaths. The cool roof and combined scenarios offer substantially larger benefits, with estimated reductions of 25.5 % (11.9 [-12.5; 35.2] fewer deaths) and 23.6 % (11.0 [-13.5; 35.3] fewer deaths), respectively. These results suggest the strong potential of urban adaptation scenarios to reduce heat-related mortality during extreme heat events.

## 4 Discussion

Our study demonstrates that heat adaptation strategies can substantially lower urban temperatures in Belgium during extreme heat events, with implications for public health and adaptation of cities to increasing heat. Our findings are in line with previous modelling studies on the cooling effect of cool roofs, which consistently show that cool roofs can lower urban air temperatures, with effects varying by city and heat event. For example, simulations in Berlin (Wang et al., 2022) and London (Simpson et al., 2024) indicate reductions in daytime average temperature of 0.7 °C–0.8 °C with cool roofs. The literature review by Krayenhoff et al. (2021) found a decrease of 0.2 °C–0.6 °C in average daily temperature per 0.1 change in albedo, placing ours (0.9 °C for maximum and 0.4 °C for minimum per 0.1 change in albedo) at the upper limit of this range. Differences can be explained by the different models and implemented albedos to represent cool roofs. Additionally, green infrastructure shows an even wider range of potential temperature reductions (0.3 °C–5 °C, Azmeer et al., 2024, Knight et al., 2021), but these outcomes depend strongly on vegetation type, spatial extent and model resolution.

Our LCZ-based analysis shows that urban morphology strongly influences both air temperature and the effectiveness of adaptation strategies, underscoring the importance of context-specific intervention planning. For example, our choice of a 20 % relative increase in urban greenery was based on current policy recommendations, but led to very limited cooling in compact urban areas. By implementing the relative change, rather than the effect of vegetation itself, we emphasise areas with intermediate levels of greenery. In line with the findings of Rahmani & Sharifi (2025), compact urban areas (LCZ2 and LCZ3) consistently experienced the highest temperatures, while open urban areas had lower baseline temperatures (Supplementary Table S4). Cool roofs provided the largest reduction in maximum temperature across all LCZs, with particularly strong effects in compact urban areas where roof coverage is high. This finding aligns with Macintyre & Heaviside (2019), who observed that cool roofs have the largest impact in areas of high building density. Similar to our findings, Jiang et al. (2025) concluded that the effectiveness of different adaptation strategies strongly depends on urban morphology.

Our rural simulations showed differences in weather at large scales via changes in, for example cloud formation and wind speed as shown in Supplementary Fig. S5. Both led to greater decreases in temperature than initially expected, but are explainable through the changes in the different variables. While the rural scenario was intended to represent changes in the local climate caused by the presence of urban areas, this shows that the impact of cities goes well beyond their physical boundaries.

Correspondingly, we estimate that heat-related mortality could have been reduced by up to 25.5 % by cool roofs, highlighting the life-saving potential of adaptation strategies. Our results are in line with the findings of Macintyre & Heaviside (2019) who found that cool roofs could reduce up to 25 % of heat-related deaths during heatwaves in the West Midlands, UK. Furthermore, having modelled cool roofs over London for the summer of 2018, Simpson et al. (2024) found a reduction in heat-related mortality of 32 %. Both studies incorporated the daily mean temperature whereas the daily maximum temperature was used in the present study. This can explain the differences in the impact of interventions on heat-related mortality reductions in both studies.

Focusing on the maximum temperature to assess heat-related mortality captures daytime heat stress, but excludes the impact of higher nighttime temperatures, which are known to impair sleep and physiological recovery (Obradovich et al., 2017, Royé et al., 2021). The choice of the temperature-mortality relationship is therefore critical: investigating the impact of the relative effectiveness of interventions by using temperature-mortality relationships based on another heat indicator, such as the daily mean temperature, as well as developing context-specific temperature-mortality relationships that account for urban morphology warrants further studies.

This study has some limitations worth acknowledging. We assumed full implementation of each adaptation strategy, with an albedo of 0.85 being the state-of-the-art in cool roofs, which may not be feasible in practice. Furthermore, we did not include a mortality analysis where age groups and gender are separated, while previous studies have shown that these population characteristics (especially age) also affect the risk of death due to extreme heat. Additionally, the mortality relation is based on outdoor maximum air temperature, which occurs when the UHI is also the lowest during the day. Gasparrini et al. (2015) used the mean daily temperature to account for both. Furthermore, by assuming that mortality depends on outdoor temperature, we neglect the benefits of indoor cooling. Minimum temperature reductions were greatest under the combined scenario, suggesting that interventions targeting nighttime cooling could yield additional health benefits. However, here we are limited by the temperature-mortality relationship and the climate model.

Future research could incorporate a cost-effectiveness analysis, thereby focussing on the feasibility of the different strategies in specific urban contexts, thus providing additional guidance to urban planning policies. Here, temperature-mortality relationships for other, multivariate heat stress variables such as the Universal Thermal Comfort Index could lead to additional valuable insights in the impact of different adaptation strategies as they account for other variations in variables that are impacted by the adaptation strategies, as discussed in Jiang et al. (2025). Following previous studies by Heaviside et al. (2016) and Simpson et al. (2024) population characteristics such as age and gender should be included as well. Continuing on our findings, temperature-mortality relationships that account for urban morphology through LCZs can be explored. Not only would this tackle the differences in urban morphology, but it would also account for differences in mortality rates between LCZs. Additionally, similar analyses that use the same approach for different cities would lead to valuable additional insights on the impact of the background climate and differences in the urban structure. Moreover, performing the same case study but with other models would allow us to assess the effectiveness of the different strategies under different urban parameterisations. Lastly, following our preliminary work (Ramon et al., 2025, Serras et al., 2024), more research focussing on future heat extremes is recommended. Overall, the different insights would provide city planners with more detailed, future-proof information that can contribute to the prioritisation of adaptations in certain urban areas as well as highlight differences between the adaptation strategies related to the urban morphology.

## 5 Conclusions

We conclude that cool roofs were the most effective intervention for lowering the daytime maximum air temperatures compared to relatively increasing the green fraction by 20 % or combining both in Belgium during the July 2019 heatwave. Combining adaptation strategies (cool roofs and more green) yielded the largest nighttime cooling. Translating these temperature reductions into health outcomes for Brussels, we estimate that up to one quarter of heat-related deaths could have been prevented if adaptation strategies had been in place in Brussels. Previous studies have highlighted the cooling potential of various adaptation strategies; however, we provide new insights into the influence of urban morphologies on the effectiveness of these strategies. Here, we found large differences between Local Climate Zones and, therefore in population exposure. While our results support existing findings on urban heat adaptation, we demonstrate that current policies should take into account the characteristics of the built environment. Lastly, we underscore the potential of heat adaptation to protect urban populations and generate broader societal and economic benefits.

## CRediT authorship contribution statement

**Fien Serras:** Writing – review & editing, Writing – original draft, Visualization, Investigation, Formal analysis, Conceptualization. **Oscar Brousse:** Writing – review & editing, Visualization, Investigation, Conceptualization. **Charles H. Simpson:** Writing – review & editing, Visualization, Investigation, Conceptualization. **Inne Vanderkelen:** Writing – review & editing, Visualization. **Claire Demoury:** Writing – review & editing, Software. **Dirk Lauwaet:** Writing – review & editing, Conceptualization. **Nicole P.M. van Lipzig:** Writing – review & editing, Conceptualization. **Clare Heaviside:** Writing – review & editing, Investigation, Conceptualization.

## Declaration of competing interest

The authors declare that they have no known competing financial interests or personal relationships that could have appeared to influence the work reported in this paper.

## Data Availability

The authors do not have the permission to share the mortality data. The data of the model simulations and scripts used for the analyses will be shared upon request after acceptance.
