# Supplementary material for: Impacts of urban adaptation on reducing temperatures and heat-related deaths in Belgium
Source: Environ Int. 2026 Jan;207:110039. doi: 10.1016/j.envint.2025.110039 (PMC12819367; doi:10.1016/j.envint.2025.110039)
Supplement: Supplementary Data 1 [file mmc1.docx]

Supplementary material of ‘Impacts of urban adaptation on reducing temperatures and heat-related deaths in Belgium’

# Supplementary Material

Supplementary Figures
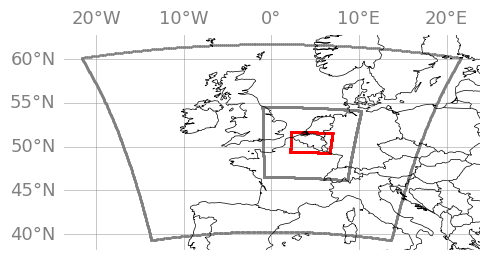


Figure S1: **Overview of the simulation domains**, with the domain used in our study indicated in red


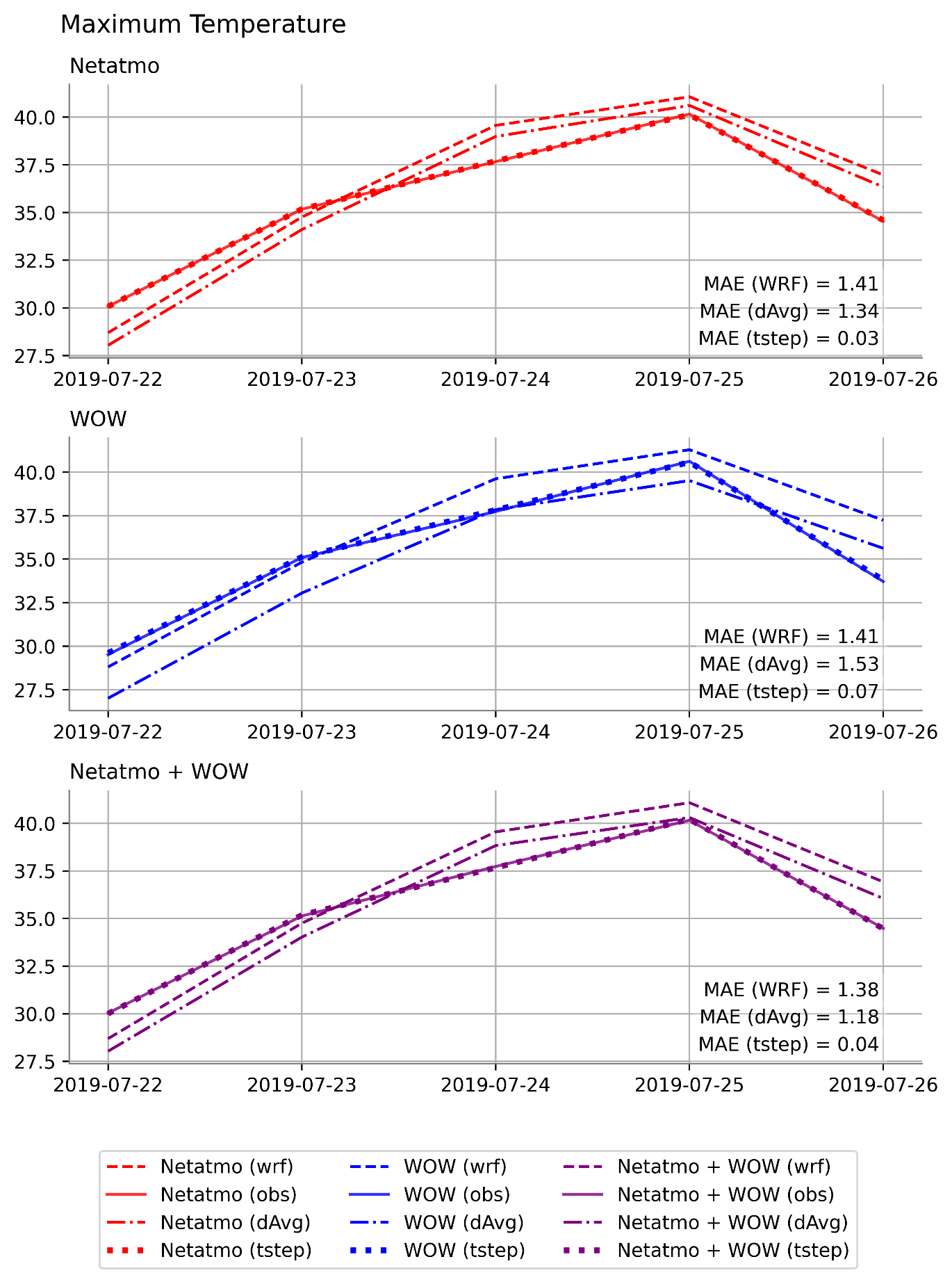


Figure S2: **Spatially and daily averaged maximum temperature**, with the daily maximum temperature being spatially averaged, for the original output (“WRF”), the observations used for bias correction (Netatmo, WOW or Netatmo and WOW) and the bias corrected temperatures (based on the daily average correction “dAvg”, or the timestep dependent correction “tstep”). The Mean Absolute Error(MAE) for the original output and the corrected temperatures is included


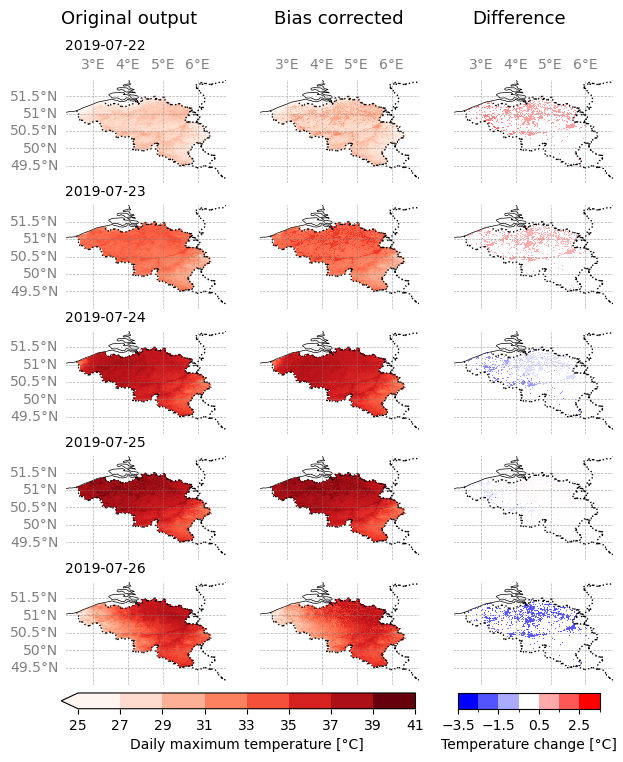


Figure S3: **Daily maximum temperatures, before and after bias correction**. The difference is calculated as 'Bias corrected - Original output'


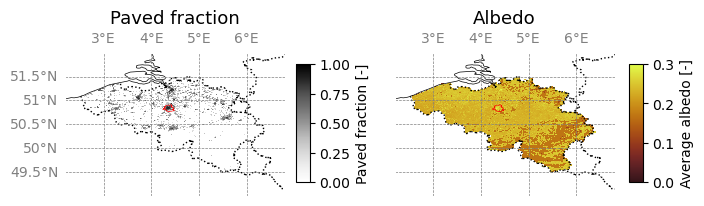


Figure S4**: Overview of the average albedo and the paved fraction** during the heatwave period under the Baseline scenario. Brussels is indicated in red


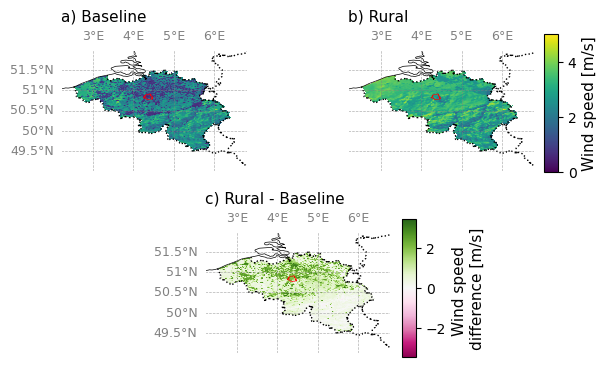


Figure S5: **Average daily wind speed for the Baseline and Rural scenarios**, as well as the difference between both. Brussels is indicated in red


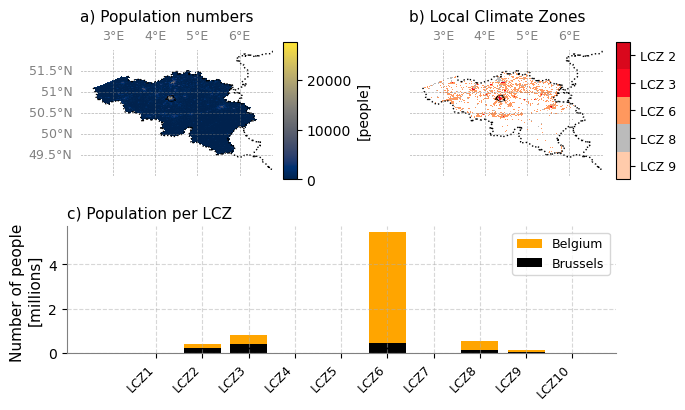


Figure S6: **Distribution of the population and Local Climate Zones** in Brussels (indicated in black) and Belgium

Supplementary Tables

Table S1: **Morphology characteristics** of Local Climate Zones in the Baseline scenario

| Local Climate Zone | Paved fraction | Roof albedo |
| --- | --- | --- |
| LCZ1 | 0.95 | 0.13 |
| LCZ2 | 0.9 | 0.18 |
| LCZ3 | 0.85 | 0.15 |
| LCZ4 | 0.65 | 0.13 |
| LCZ5 | 0.7 | 0.13 |
| LCZ6 | 0.6 | 0.13 |
| LCZ7 | 0.85 | 0.15 |
| LCZ8 | 0.3 | 0.18 |
| LCZ9 | 0.55 | 0.13 |
| LCZ10 | 1.00 | 0.1 |

Table S2: **Average Residuals after Bias Correction**. 3 types of observation datasets (Netatmo, WOW and both combined) were combined with 4 types of regressors (Linear regression, Ridge, Lasso and Random Forest). The training dataset was optimised for the RMSE, and the test dataset was evaluated with the MEA and the coefficient of Determination (R²).

| Observations | Model | mean RMSE (training) | Mean MAE (testing) | mean R² (testing) |
| --- | --- | --- | --- | --- |
| Netatmo | Linear | -2.03 | -1.64 | -0.01 |
|  | Ridge | -2.03 | -1.65 | -0.02 |
|  | Lasso | -2.06 | -1.65 | -0.02 |
|  | Random Forest | -1.80 | -1.68 | -0.06 |
| WOW | Linear | -0.88 | -0.75 | -0.45 |
|  | Ridge | -0.79 | -0.71 | -0.30 |
|  | Lasso | -0.90 | -0.74 | -0.42 |
|  | Random Forest | -0.67 | -0.64 | -0.03 |
| NETATMO + WOW | Linear | -1.96 | -1.57 | -0.00 |
|  | Ridge | -1.96 | -1.57 | 0.00 |
|  | Lasso | -1.99 | -1.57 | -0.01 |
|  | Random Forest | -1.73 | -1.59 | -0.04 |

Table S3: **Average daily minimum and maximum temperatures** for the baseline scenario for the 1st, 50th and 99th percentile of the population

| Fraction of the population | Daily Average minimum temperature | Daily Average Maximum temperature |
| --- | --- | --- |
| 1% | 19.1°C | 33.1°C |
| 50% | 22.4°C | 36.4°C |
| 99% | 23.8°C | 37.3°C |

Table S4: **Average daily temperatures** across different LCZs for the study period

| Local Climate Zone | Daily Average minimum temperature | Daily Average mean temperature | Daily Average Maximum temperature |
| --- | --- | --- | --- |
| LCZ1 | - | - | - |
| LCZ2 | 21.99°C | 28.21°C | 34.30°C |
| LCZ3 | 21.58°C | 28.10°C | 34.51°C |
| LCZ4 | - | - | - |
| LCZ5 | - | - | - |
| LCZ6 | 20.63°C | 27.47°C | 34.18°C |
| LCZ7 | - | - | - |
| LCZ8 | 20.15°C | 26.93°C | 33.72°C |
| LCZ9 | 20.05°C | 26.96°C | 33.87°C |
| LCZ10 | - | - | - |
